# Remarkable Case of Placenta Prævia

**Published:** 1868-08-01

**Authors:** Hiram Wanzer

**Affiliations:** Chicago, Ill.


### REMARKABLE CASE OF PLACENTA PRÆVIA,

*Spontaneous Delivery and Recovery. Related to the Chicago Medical Society, with pathological specimen presented. By* HIRAM WANZER, M.D., *Chicago, Ill.*

MR. PRESIDENT AND GENTLEMEN:—I have a pathological specimen—a fœtus, at about six and a half months, for the examination of the society this evening. Having not been in attendance, nor an inspector of the parturient process, I am obliged to the family for my data in the antecedents of the case.

I was called on the 19th of April ult., at 8 o'clock A. M., to Mrs. B——, an American, aged 23, who, just previous to my arrival, had given birth to this fœtus. She stated that her previous health had been good; that she had been married eight years, and given birth to three children; that her previous gestations and the delivery were natural; that nothing abnormal presented in this case, until eight weeks previous, when she began flowing, and during that time she had lost daily as much blood per vaginam, as she was accustomed during her catamenial period; that eight days previous, she began to have some labor pains, commencing at about noon and disappearing at midnight, when there was quietude obtained; that the periodicity continued in this manner for four days, when more active pains supervened, recurring frequently and with greater severity until the morning of its birth. The hæmorrhage being the same in quantity during her parturient efforts as before.

The second day previous to my being summoned, a German midwife was called, who has informed me that the os uteri was rigid and undilated. Her services being dispensed with, an intelligent physician was sent for, who administered *Morphia*, in order that she might procure some rest from her long fatigue. He states that there was a degree of rigidity of the os at the time, and that it only dilated to the size of a three cent piece; that the pains were considered inefficient, and of a neuralgic character. I found the following morning she had given birth to this fœtus, as previously stated, with the membranes entire. It was a breech presentation; the placenta, as you see, is firmly attached by the arterial and venous radicles of its structure with the chorion, excepting its free peripheral border, where it is slightly detached. This firm coalescence extends over the nates of the child, on the right side as far as the shoulder, also on left side as far as the crest of the illium. The maternal surface of the placenta denuded of the membranes, has a fleshy look, and is divided into numerous sulci or small lobes. Its extensive surface of attachment to the mucus membranes of the neck body, and a portion of the fundus of the uterus. On the right side, expanding by the physiological growth of the child, and with the regular development of the uterus, accounts for its thinness, and in part for the anomaly it presents. Will the society explain how far the specimen is pathological, and the cause of such expansion, and extensive surface of attachment? Not only rare, as I believe, and interesting as it is, might it not have arisen from the abnormal shortening and traction of the umbilical cord, and through the diaphanous membranes, where, covered by the placenta, are seen the fœtus *in situ*

floating in the amniotic fluid with the cord (tense) around its neck. The mother states that she felt motion in the child from the time she began flowing to the time of its birth. The patient, at the time of my arrival was flowing considerably, which completely subsided under the administration of *Tinct. Ergot*, pressure over the uterus, and moderately tight bandage. Her countenance wore a somewhat blanched appearance, but there was not that degree of anemia we might anticipate from such prolonged hæmorrhage. After sufficient contractility of the uterus was secured, I administered every four hours, 20 gtt. *Tinct. Ferri Muriatis*, alternated with wine and nutritious diet. On the fifth day she was convalescent, and able to attend to her household duties. Authors, both ancient and modern, have agreed that among all the causes which make labor difficult, there are are none fraught with so much peril to the mother as placenta prævia. The ratio of maternal mortality being even greater than the two most fatal epidemics of yellow fever and Asiatic cholera, and more than twice that of lithotomy.

Gottleib Thacher, professor, of Leipsic, wrote a dissertation in 1709. One of his observations was upon the death of a woman from flooding, at the end of pregnancy. The autopsy revealed that the placenta was attached to the cervical portion, and closed the os uteri. The membranes were unbroken, and intimately connected with the whole of the internal surface of the uterus.

Dionis, who wrote in 1721, while recognizing the fact that the placenta is often found presenting the os uteri, controverts the opinions of Mauriceau, that its detachment was caused by the shortening of the cord.

Lemotte says there are none more perilous than that in which the afterbirth presents before the child.

Hæmorrhage, says Delneyre, is a fearful occurrence to a woman. It may be slight or considerable, dependant upon the partial or total detachment of the placenta during convulsions, with which the mother may be attacked, or from the attachment of the placenta upon the os.

Conquest says that hæmorrhage from this cause, places the woman in the most imminent danger.

Dr. Collins says the attachment of the placenta to the mouth of the womb, is one of the most dangerous complications to be met with in the practice of midwifery.

Dr. John F. Ramsbotham says, a woman placed in this perilous situation, therefore, holds her life under a very uncertain tenure.

Mr. I. Ingleby remarks that the patient is necessarily exposed to danger of a peculiar kind, imminent in degree, involving the deepest responsibility, and demanding the exercise of the highest judgment.

Madam La Chappelle remarks, for these reasons it follows that hæmorrhage, which depends upon the adherence of the placenta to the internal orifice of the uterus, is one of the most dangerous accidents to which women are exposed during their pregnancy.

The able writings of Cazeau, Dr. F. Churchill, and Prof. C. D. Meigs corroborate the opinions of those distinguished writers mentioned.

I will not enter this evening upon the pathology and treatment of this most appalling accident in the parturient woman, as each case has not only its own specific type, but also its indications for treatment. I will simply confine myself to the case reported, and I accord fully with the testimony left us by those eminent men mentioned. The question suggests itself, had we the early management of the case, would it have been prudent to expedite the delivery, or leave her as she was to the unassisted powers of nature, which managed the case so well without our interference. As before stated, the hæmorrhage never exceeded her daily catamenial loss. The patient being robust and of unusual constitutional vigor, under the circumstances, I think non-interference was strongly indicated. The extensive surface occupied by the placenta would have been a barrier, in rupturing the membranes and delivery by version without perforating them, which would have been no easy matter, bound as it was to them by the placental vessels; and, furthermore, it would have exasperated the hæmorrhage. The hæmorrhage, no doubt, occurred from the gradual and partial detachment of the placenta from the uterine walls. The want of the dilatation of the os, the physician states, was fourteen hours previous closed and rigid. It was in consequence of the firm attachment of the placenta to the os, hermetically sealing it, except at the point whence the hæmorrhage proceeded. May not this have been conservative, acting as a tampon for the prevention of an exhausting hæmorrhage, until the complete detachment of the placenta, the dilatation of the os, and lastly the expulsion of the fœtus? I think cases of spontaneous delivery in placenta prævia are very infrequent.

